# Evaluation of the CorInnova Heart Assist Device in an Acute Heart Failure Model

**DOI:** 10.1007/s12265-018-9854-5

**Published:** 2019-01-02

**Authors:** Erica C. Hord, Christina M. Bolch, Egemen Tuzun, William E. Cohn, Boris Leschinsky, John C. Criscione

**Affiliations:** 1 Department of Biomedical Engineering, Texas A&M University, Mailstop 3120, College Station, TX 77843-3120 USA; 2 CorInnova, Incorporated, Houston, TX USA; 3 Texas A&M Institute for Preclinical Studies, Texas A&M University, College Station, TX USA; 4 Texas Heart Institute, Houston, TX USA

**Keywords:** CorInnova, Heart assist, Direct cardiac compression, Temporary support, Acute failure, Cardiac devices, Cardiac unloading

## Abstract

While the number of patients supported with temporary cardiac assist is growing, the existing devices are limited by a multitude of complications, mostly related to contact with the blood. The CorInnova epicardial compressive heart assist device was tested in six sheep using an acute heart failure model. High esmolol dose, targeting a 50% reduction in CO from healthy baseline, resulted in a failure state with mean CO 1.9 L/min. Heart assist with the device during failure state resulted in an average absolute increase in CO of 1.0 L/min, along with a decline in ventricular work to 67.5% of the total LV SW. Combined with repeated success of minimally invasive device implant, the resulting increases in cardiac hemodynamics achieved while still unloading the heart demonstrate the potential of the CorInnova device for temporary heart assist.

**Electronic supplementary material:**

The online version of this article (10.1007/s12265-018-9854-5) contains supplementary material, which is available to authorized users.

## Introduction

An estimated 6.5 million people in the USA alone have heart failure (HF) in some form, with an estimated 26 million people worldwide. This US population is projected to increase to 8 million by 2030, as the incidence is increasing due to aging of the population and increased survival of heart attacks [1]. A growing number of these patients need short-term, bridge-to-decision (BTD) circulatory support while a more permanent treatment strategy is decided upon. BTD has the potential to increase survival outcomes in patients bridged to longer-term mechanical circulatory support (MCS) devices by stabilizing hemodynamics and improving end-organ function, thus reducing patients’ surgical risk prior to more permanent device implantation. However, current BTD devices contact the blood, introducing a multitude of potential complications [2]. CorInnova’s technology aims to provide a safer, more effective, and more intentional BTD device.

The CorInnova device is a cardiac assist device in preclinical development that applies compression to the outside of the heart (epicardium) in synchrony with systole. The primary advantage of the CorInnova device is the non-blood contacting mode of heart assist. All current temporary MCS devices—including intra-aortic balloon pump (IABP), extracorporeal membrane oxygenation (ECMO), CentriMag, TandemHeart, and Impella—have the potential to cause vascular injury, bleeding, and neurologic injury complications [2]. Additionally, the CorInnova device is non-obligatory and can be turned off as needed. This feature provides the potential to wean patients off the pump during signs of improved status. Finally, the mode of assist is synchronized with the native heartbeat and designed to preserve the native heart motion, guiding healthy growth and remodeling while unloading the heart.

As discussed by Moreno et al., a direct cardiac compression (DCC) device that is strategically designed to correct aberrant heart motions and enhance heart contractility may provide both heart assist and a potential means to enable heart recovery—provided that proper deformation of myocardium is needed for heart tissue growth and remodeling [3, 4]. The results in the Moreno single animal pilot studies demonstrate the possibility of restoring proper cardiac hemodynamics and kinematics with a soft DCC device implanted through a less invasive procedure than standard open-heart surgery. This report furthers this work with design improvements and with multiple studies in an ovine model of acute heart failure.

## Methods

### CorInnova Cardiac Assist Device

**Fig. 1:**
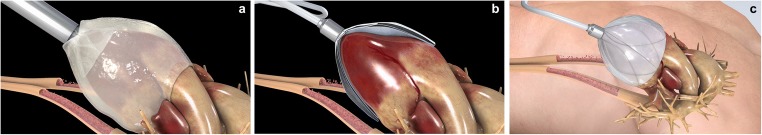
Illustrations of the CorInnova heart assist device. The device is deployed inside the pericardial sac (**a**); the device consists of an inner (epicardial) fluid-filled polyurethane film buffering component and an outer polyurethane film active assist component (**b**); a Nitinol frame provides self-expanding capabilities (**c**)

The CorInnova device is situated within the pericardium and surrounds both ventricles. The device encloses most of the LV free wall and a majority of the RV free wall from the apex to the atrioventricular (AV) groove. Ventricular coverage is limited by the insertion point of the inferior vena cava (IVC) into the right atrium, and therefore resides apical to the AV groove. The device consists of two concentric sets of thin film polyurethane bladders—an inner (epicardial) fluid-filled buffering component that accommodates any gaps in the interface between the device and heart; an outer bladder component is cycled to inflate with air in synchrony with the native heartbeat, providing active epicardial compression during systole consistent with physiologic cardiac motion. A superelastic Nitinol frame gives the device structure, while enabling minimally invasive self-deployment and diastolic recoil. See Fig. 1 for an illustration of the device components.

The device was operated using a custom programmable pneumatic drive system—referred to as the Driver. The ovine ECG was acquired using epicardial electrodes and was gated on the R-wave by a custom-gain cardiac trigger monitor (Model 7700, IVY Biomedical). Using a programmable controller, the R-wave gated ECG signal is used to trigger the activation of solenoid valves, which pressurize the active bladders during systole, and vacuum during diastole. A Millar Mikro-Tip® Catheter Pressure Transducer (Model SPR-320, Size 2F) was used to measure the fluid pressure in the passive chambers, providing a metric of epicardial assist pressure. The device was activated for short periods of time (< 5 min) during this study with intermittent periods of device standby to observe the relative acute effects of device assist.

### Study Design and Surgical Procedure

Six (6) separate acute ovine, non-GLP studies were conducted for this investigation, with two excluded from data analysis due to suboptimal device fit around the heart. All experiments were conducted under an animal use protocol (AUP) that was approved by the Texas A&M University Institutional Animal Care and Use Committee (IACUC) or the Texas Heart Institute IACUC. Each study utilized a single, anesthetized adult domestic cross ovine (50–78 kg). Upon induction of anesthesia, catheters were placed to facilitate the administration of medications as well as for monitoring of vital parameters. Central venous pressure (CVP), aortic pressure (AoP), and left ventricular pressure (LVP) were monitored by either fluid-filled catheters or a Millar Mikro-Tip® Catheter Dual Pressure Transducer (SPR-751) placed across the aortic valve. A left thoracotomy was performed to access the base of the heart, where a small incision was made in the pericardium to expose the aortic root for placement of a Transonic COnfidence Flowprobe® around the ascending aorta to measure cardiac output (CO) before closing.

An apical approach to the heart was achieved through a substernal incision with removal of a portion of the xyphoid process. After performing the xyphoidectomy, the surgeon opened a small (approximately 1 in. diameter) incision at the pericardial apex, and then constructed a pericardial cradle to stabilize the pericardial edges. A pericardiogram was performed for imaging of the epicardial structures by infusing contrast media directly into the pericardial sac through the apical incision [5]. Prior to deployment, the device was compressed into a cylindrical deployment tube (PTFE 2.5 cm OD, 2.3 cm ID, 20 cm length), with radiopaque marker tape used to visualize the end of the tube under fluoroscopic imaging (Indicator® 1.0 mm Lines–IZI Medical Products). To prepare for implantation, the deployment tube was placed inside the pericardial cradle to align the fluoroscopy C-arm with the plane of the device. The device was then advanced from the deployment tube around the heart under fluoroscopic guidance, in a self-expanding fashion inside the pericardium [6]. Once the device was in place around the heart (with positioning verified by fluoroscopy), the deployment tube was retracted, and the pneumatic driveline was routed to exit through the substernal incision before closing. After removing air in the chest, the passive bladder of the device was filled with an appropriate volume of imaging contrast media such that the pressure measured in the passive chambers during device standby reflected the left ventricular filling pressure. Finally, after filling the passive chamber and connecting the epicardial electrodes, assist was initiated at a low pressure to confirm stable ECG R-wave gating.

A high dose of esmolol was utilized to create an acute model of a heart with significantly depressed function. In addition to creating a stable, dose-dependent failure, esmolol has the benefit of rapid metabolism with a clearance half-life of only 9 min—allowing for rapid return to the baseline state [7]. After all measurements were collected during the animal’s baseline state, an appropriate combination of bolus (20–150 mg) and continuous rate of infusion (CRI) (10–300 mL/h, [10 mg/mL]) was administered to target a 50% reduction in CO from baseline. The dose varied with each animal, and was managed by the animal anesthesia staff. The device was activated upon > 20% reduction in CO from baseline; the animals were not in failure without assist, other than to collect device standby samples, for reference.

### Data Collection and Statistical Analysis

ADInstruments PowerLab data acquisition hardware and LabChart software were used to continuously record all physiologic parameters and CorInnova device data. Paired samples of 20 s of device standby and successive device assist were collected for analysis of acute effects; the duration was selected in order to average out pressure artefact due to anesthesia respirations by including two full respiration cycles in the sample (mean *n* = 31 ± 3 cardiac cycles per 20-s sample). The samples of cardiac cycles were averaged over the 20-s selection using LabChart, and then averaged in Microsoft Excel to provide an overall effect for each animal as well as a comprehensive trend. A two-tailed, paired *t* test was used for comparison between device standby and assist for each parameter, with an alpha level of 0.05 (Excel).

## Results

### Deployment

**Fig. 2:**
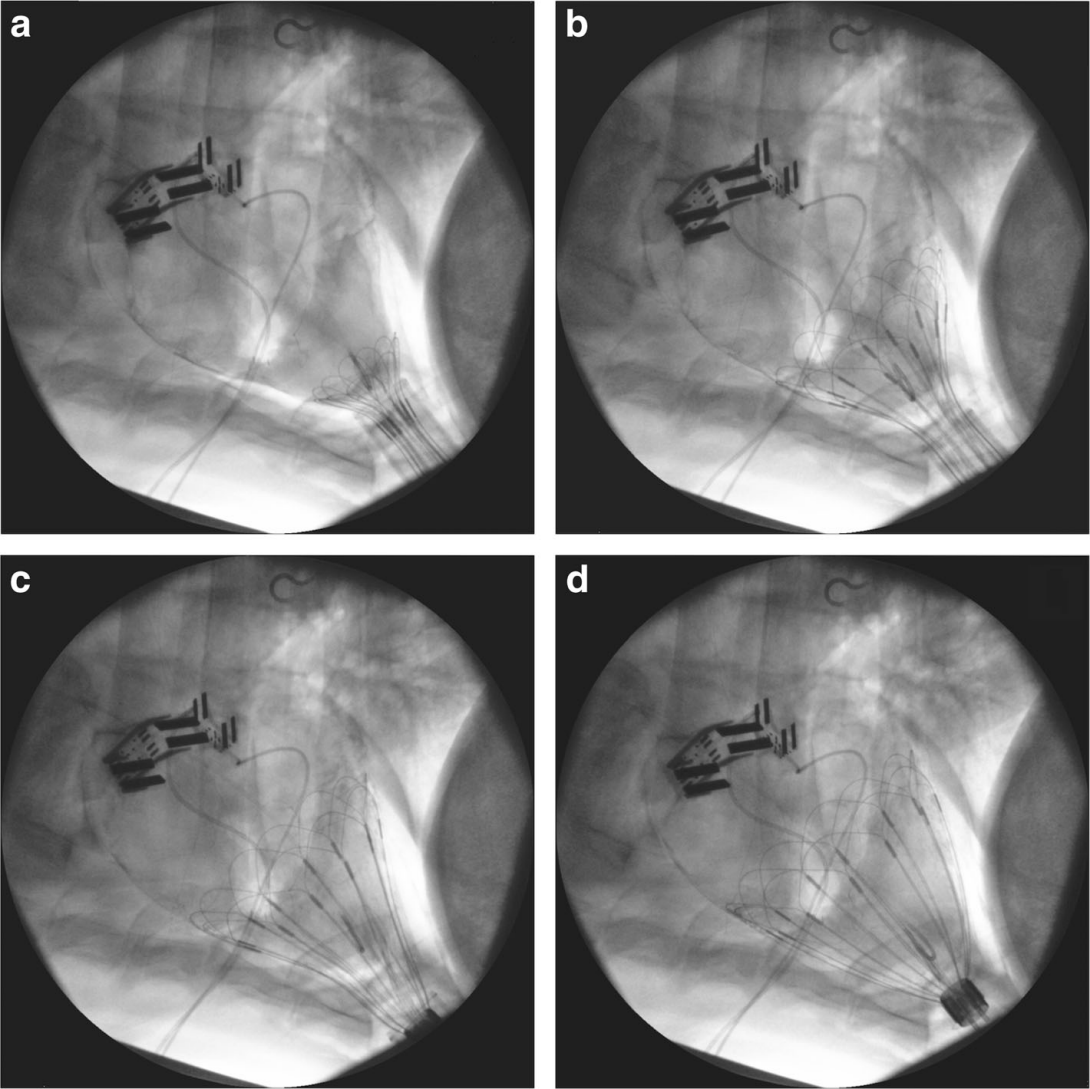
Fluoroscopic imaging of minimally invasive deployment of the CorInnova heart assist device; initial advancement from the deployment tube, inside the apex of the pericardium (**a**), progressive stages of deployment (**b**, **c**), and the device in place around the heart, inside the pericardium (**d**)

Minimally invasive deployment was successful and uneventful on the first attempt for five out of the six studies and was successful on the second attempt for the remaining study. In each case, device placement was completed without the need for cardiopulmonary bypass and with placement time after initial advancement to removal of deployment tube on average less than 25 s. Figure 2 shows the stages of minimally invasive, self-expanding deployment inside the pericardial sac under fluoroscopic imaging guidance. A video of this deployment process collected in vivo has been included as supplementary material. Note from Fig. 2d that the fully deployed device does not extend above the AV groove.

### Acute Failure Assist

**Fig. 3:**
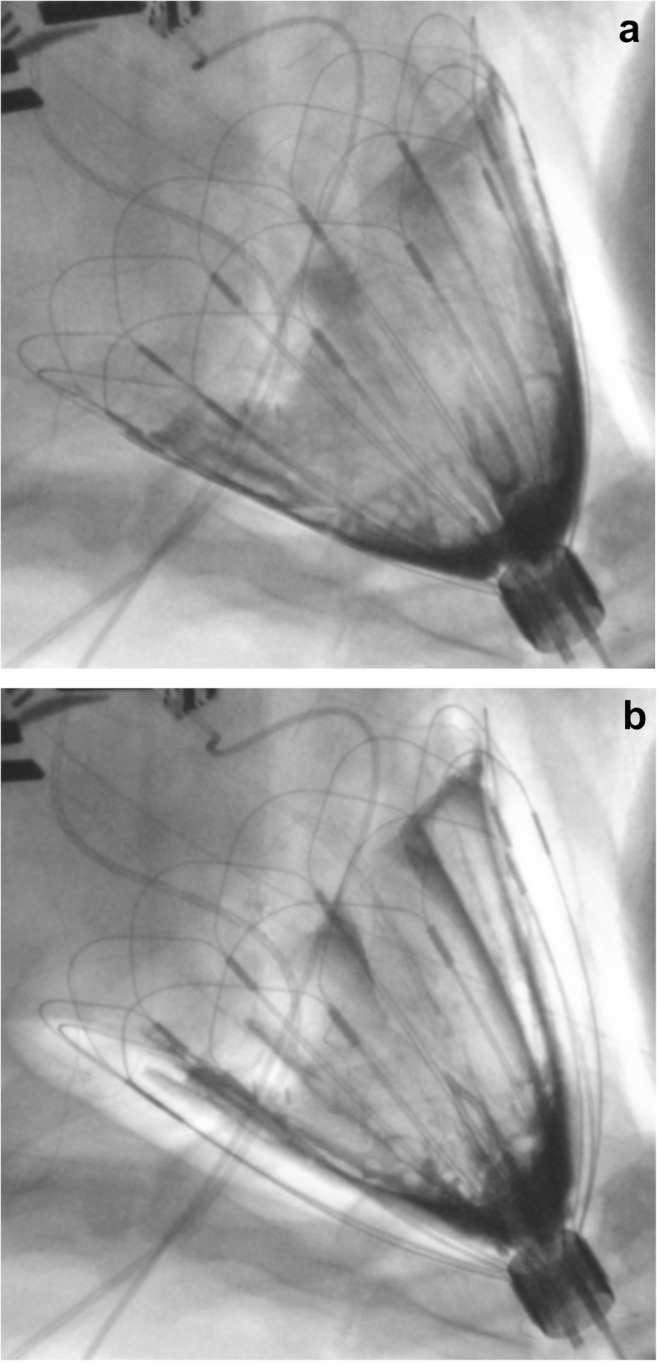
Fluoroscopic images of the device during diastole (deflated) (**a**) and during systolic assist (inflated) (**b**); imaging contrast media was used as the fluid in the passive bladder for visual aid (darkest regions)

**Fig. 4:**
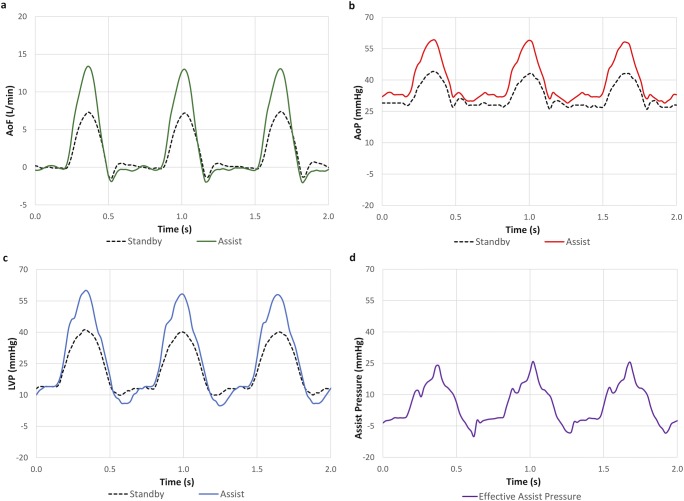
Representative hemodynamic waveforms sampled from the in vivo data; the dotted black line shows the unassisted waveform, and the colored lines indicate the hemodynamic waveforms during assist; aortic flow (**a**), aortic pressure (**b**), left ventricular pressure (**c**), and effective assist pressure (**d**)

Upon activating the device on the failure heart, several hemodynamic parameters showed trends of marked improvement with systolic assist. The average changes with assist relative to the failure state hemodynamic data, as well as the baseline hemodynamics are summarized in Table 1. In terms of recovering the healthy baseline prior to esmolol failure state, device assist recovered the CO by an average of 74% and the LV stroke volume (LV SV) by 86% (mean of 4 animals). Figure 3 shows fluoroscopic cine capture of the device during standby (Fig. 3a) and during assist (Fig. 3b). Figure 4 shows representative hemodynamic waveforms of device standby and assist during failure state.

**Table 1:** Changes in mean hemodynamics with assist. Summary of mean hemodynamic changes with device assist during esmolol failure averaged over four acute ovine studies, compared to the failure state and baseline; each sample was 20 s of cardiac cycles; *p < 0.05

	HR	CO	LV SV	SBP	DBP	MAP	LVP	CVP	LV EDP	LV SW	Mean systolic assist pressure
	BPM	L/min	mL	mmHg	mmHg	mmHg	mmHg	mmHg	mmHg	mmHg*mL	mmHg
Baseline	*108 ± 14*	*4.1 ± 1.0*	*38 ± 9*	*82 ± 6*	*60 ± 10*	*69 ± 9*	*83 ± 10*	*10 ± 3*	*13 ± 3*	*2813 ± 356*	
Mean failure state	*90 ± 3*	*1.9 ± 0.2*	*21 ± 3*	*50 ± 3*	*31 ± 4*	*38 ± 4*	*47 ± 6*	*14 ± 2*	*16 ± 3*	*955 ± 61*	
Mean with assist	*91 ± 3*	2.9 ± 0.4*	32 ± 5*	65 ± 5*	*32 ± 8*	47 ± 6*	63 ± 9*	*13 ± 2*	*15 ± 2*	1924 ± 129*	*17 ± 5*
Absolute change with assist	*1 ± 1*	*1.0 ± 0.2*	*11 ± 2*	*16 ± 3*	*0 ± 7*	*9 ± 2*	*16 ± 4*	*− 1 ± 1*	*− 1 ± 2*	*968 ± 132*	*17 ± 5*

**Fig. 5:**
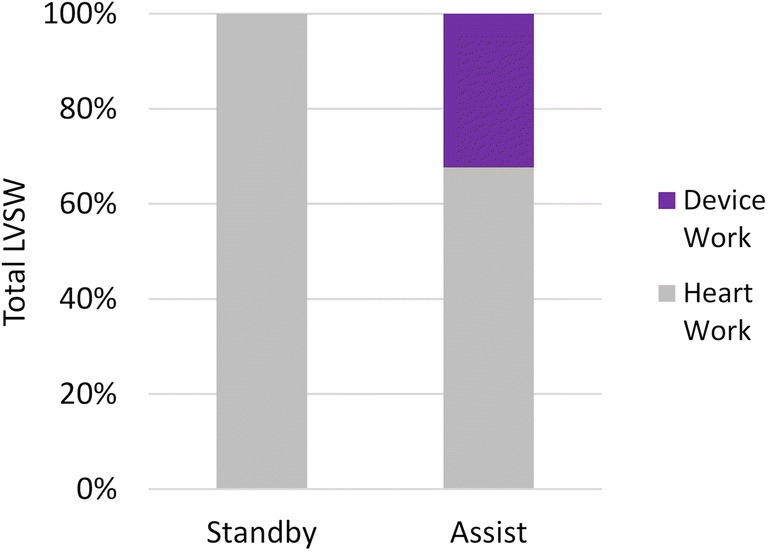
Illustration of the decline in the work done by the heart relative to the total LV SW during device assist

Overall, the CO increased by 1.0 ± 0.2 L/min and the LV stroke work (LV SW) increased by 968 ± 132 mmHg*mL (+ 102%). Moreover, the work due to the device during assist (*W*_D_) was subtracted off the total LV SW to quantify the percentage of the total LV SW due to endogenous ventricular work (*W*_LV_); overall, while the LV SW during device standby was 100% due to the work done by the heart (*W*_LV_), during device assist, the average *W*_LV_ was responsible for only 67.5% of the total LV SW, with *W*_D_ responsible for the remaining 32.5% of LV SW. This metric of LV unloading is plotted in Fig. 5.

With an average device assist pressure of 17 ± 5 mmHg, systolic blood pressure (SBP) improved by 16 ± 3 mmHg, and similarly, the peak LVP improved by 16 ± 4 mmHg with assist. Venous return pressures showed minimal change; the overall CVP decreased by 1 ± 1 mmHg, and the LV end-diastolic pressure (LV EDP) decreased by 1 ± 2 mmHg).

## Discussion

This study demonstrated the acute effects of the CorInnova heart assist device in an ovine model of acute heart failure, building on the pilot investigation of the device described in detail previously by Moreno et al. The repeated success of minimally invasive deployment on the first attempt shows significant confidence in the practical implantation and implementation of this device in the clinical setting. Moreover, the device was implanted at two different study locations with different surgical teams, further demonstrating the ease of use of this technology. While a subxyphoid incision is the best surgical approach to access the apex of the heart in the ovine anatomy, a mini-left thoracotomy would be more appropriate for an apical approach for implantation of this device in humans. It is anticipated by author WEC, a cardiothoracic surgeon who performed the animal implant, that the total skin-to-assist time would be less than 10 min in an acute clinical setting. Given that many patients with heart failure are considered high risk for major surgery, a minimally invasive device placement procedure is advantageous; moreover, the procedure avoids a median sternotomy, which may be later required when bridging to implant a long-term ventricular assist device (VAD). The swift deployment time—as quick as 15 s after access to the pericardial sac is established—opens up opportunities for other applications of this technology, such as in cases of acute cardiogenic shock.

Short-term assist for BTD is a justified strategy for stabilizing a patient prior to implantation of a long-term VAD; with more stable hemodynamics and improved end-organ function, the patient has reduced risk of poor outcomes. Most importantly, if the patient can be bridged to recovery, they can avoid the unnecessary risk of major surgery, VAD complications, and the cost of an expensive VAD. All existing temporary support devices are associated with several complications, including bleeding, high risk of vascular injury, infection, and/or neurologic complications; patients who have peripheral vascular disease and contraindications to anticoagulation are not eligible for currently approved MCS device support. In contrast, the CorInnova device has the advantage of non-blood contacting biventricular (BiV) support—particularly beneficial when there is risk of thrombosis and/or right-heart failure. In summary, we anticipate that the device may be effective for a multitude of acute heart assist situations, including but not limited to cardiogenic shock, acute decompensated heart failure, myocarditis, post-cardiotomy assist, failure to wean, and for bridge-to-decision in patients with chronic heart failure.

The results of the present study demonstrated improved cardiac output and hemodynamic pressures achieved with a relatively low epicardial assist pressure. Furthermore, the work performed by the heart was reduced with device assist. Although systolic changes were consistent, the effect of device assist on the diastolic blood pressure was variable between animals and will need to be further investigated. Additionally, it is unlikely that uniform BiV epicardial compression over-assisted the right heart, where lower pressures are the norm; there was a slight decline in LV EDP with assist, showing no indication of increased LV preload due to elevated pulmonary pressures. It should be noted that only a small reduction in filling pressures was anticipated with assist in the esmolol model due to the low preload associated with this particular failure model; it is anticipated that in future studies with higher preload models of HF, this metric of cardiac unloading will have a more pronounced change with device assist in addition to the reduction in ventricular work. Overall, the decline in ventricular work combined with the slightly reduced CVP and LV diastolic pressures shows evidence that device assist unloads the heart while increasing CO. Another metric for assessing unloading is afterload, and conventionally, SBP is a good measure of afterload because the epicardial pressure is nearly zero. However, when the epicardial pressure is increased with the device assist herein, the myocardial load is reduced because the heart muscle does work against the transmural pressure difference (which is approximated as SBP minus systolic assist pressure).

### Comparison to Other Temporary MCS

The hemodynamic effects induced by the CorInnova device were on the order of several existing temporary MCS devices. The intra-aortic balloon pump (IABP) has been clinically available for several decades; however, the resulting effects on CO are modest with debatable effect on patient outcomes. A clinical study comparing the hemodynamic effects of the IABP to the Impella 2.5 reported an increase of only + 0.21 L/min from 3.46 to 3.67 L/min in the IABP group [8]. A more recent study comparing the 40 cc balloon to the newer (2012) 50 cc balloon reported slightly increased, albeit highly variable, absolute increases in CO; counterpulsation activation of the 40 cc balloon increased CO by 0.7 ± 0.9 L/min, whereas activation of the 50 cc balloon resulted in a 1.4 ± 1.0 L/min increase in CO [9]. Although CO is not always reported separately from “pump flow” before and after MCS device implantation, the beforementioned Seyfarth et al. 2008 clinical study comparing IABP with the Impella 2.5 demonstrated an increase in CO from 3.16 to 4.12 L/min (∆0.96 L/min) for the Impella 2.5 group; the observed change in CO was less than 1.0 L/min, despite maximum pump flow rates of 2.5 L/min. Although higher pump flow rates can be achieved with different Impella devices (i.e., CP/5.0/LD), it is also expected that higher CO can be achieved with the CorInnova heart assist device at higher assist pressures. Moreover, the Impella 5.0 and LD are larger and require surgical cut down of the femoral artery or axillary artery, or an open-heart procedure (Impella LD).

Also of note from the Seyfarth study was the figure showing the decline in cardiac power index (CPI) due to the contribution of the heart during the first 14 h post-implant, demonstrating cardiac unloading. This CPI due to the heart was calculated by separating the Impella pump flow from the total CO, and subtracting the contribution of the device from the total CPI. They also showed that endogenous cardiac work of Impella patients was significantly lower than in patients with IABP at all time points [8]. A similar effect was observed in the LV work calculated during this study; during assist with the CorInnova device, only 67.5% of the total LVSW was due to the endogenous LV work. This demonstrates that although the heart is stimulated to perform some ventricular work during assist, it still achieves a higher total LVSW without having to perform 100% of the augmented LVSW, and thus reducing the load on the heart.

ECMO can assist in gas exchange with some improvement in CO. However, ECMO increases the afterload on the heart. Additionally, a recent study comparing the outcomes of patients who were bridged with various temporary MCS devices found that veno-arterial ECMO (VA-ECMO) was associated with worse outcomes in LVAD patients than patients who were not bridged to LVAD [10]. This study demonstrated the possibility that VA-ECMO is not justified for use as a bridge to a long-term MCS device. In this case, the CorInnova device may be a better choice for patient BTD.

Other temporary MCS devices, such as the TandemHeart and CentriMag, report higher CO than the CorInnova device. However, the tradeoff is increased invasiveness and risk to the patient. The CentriMag pump is surgically implanted, cannulated left atria (LA) to aorta for LV support or right atria (RA) to pulmonary artery (PA) for right ventricular (RV) support; the CentriMag can also be used peripherally in an ECMO circuit. However, while the CentriMag pump itself can achieve up to 10 L/min flow, it is only approved for up to 6 h for LV support, and RV support up to 30 days under Humanitarian Use only. Similarly, the TandemHeart has the ability to pump up to 4 L/min, with bypass from the LA accessed by a venous transseptal inflow cannula, and outflow to the femoral artery. This allows for oxygenated blood to be pumped from the LA to the systemic circulation via a femoral artery catheter. While these devices have been shown to significantly reduce preload and augment CO, there is high risk of thromboembolism and thus patients receive a systemic heparin regimen for anticoagulation [2]. Given the invasive surgery, limited duration of use, and multiple complications associated with each of these pumps, the CorInnova device may be a better option for BTD—especially for patients contraindicated to anticoagulation therapy. Furthermore, while BiV assist is sometimes possible with these devices, duplicated surgical cannulation to accommodate two pumps (one for each ventricle) is required; in contrast, the CorInnova heart assist device is innately biventricular.

Heart assist devices similar in concept to the CorInnova device have been explored in the past [11]. More recently, the Soft Robot Device tested in a porcine esmolol model of acute heart failure yielded hemodynamic results very similar to the CorInnova device with an increase in CO of ~ 1.4 L/min during acute heart failure (approximated from Fig. 7c) [12]. The same group of investigators followed up this initial work with an altered design, featuring soft robotic ventricular actuator bands. This device was also tested in a porcine esmolol acute HF model, with a reported absolute change in CO of ~ 0.8 L/min (approximated from Fig. 6 and Fig. 8) [13]. In contrast to the CorInnova methods, neither the Roche et al. nor the Payne et al. study demonstrated a minimally invasive surgical approach; a sternotomy was required for device implantation and the pericardial sac was not preserved.

### Bridge to Removal

The less invasive surgical implantation of the CorInnova device combined with the intrinsic pneumatic attachment method is ideal for bridging the patient to potential device removal upon improvement in myocardial function. More specifically, the device does not require any suturing or cardiac chamber cannulation that ultimately results in myocardial damage—limiting chances of myocardial recovery. In the present acute studies, no bleeding or ecchymosis was observed when removing the device from the heart. However, while the CorInnova device is designed to minimize adhesions, it remains to be seen how easily the device can be removed at the conclusion of chronic studies. Although no universal criteria for MCS weaning to test for removal readiness currently exists, the non-obligatory nature of the CorInnova device permits the magnitude of device assist to be reduced and even halted completely without the risk of thrombogenic complications. In a recent review by Selzman et al., the authors encouraged a paradigm shift towards intentional bridging to device removal—a strategy the CorInnova device is ideal to help achieve [14].

### Study Limitations

The esmolol acute failure model, although commonly used for preclinical testing of similar devices, has limitations. A high dose of a beta-blocker, such as esmolol, is very effective in reducing the cardiac contractile strength and stroke volume. Additionally, the esmolol model is consistent with other DCC acute heart failure investigations (permitting direct comparison of study results), is low cost, has low risk of animal mortality, and fewer arrhythmias. However, esmolol causes dilation of the great vessels, resulting in significantly reduced pulse pressures and low preload. Therefore, the device needed to overcome significantly reduced preload, which may have affected the recovery of cardiac output; a low preload may have limited the CO effects of epicardial ventricular assist, and yet, a low afterload may have enhanced it. The low preload also limits the observable cardiac unloading effect of the device in regards to reducing filling pressures and will need to be studied in a higher preload HF preclinical model. Additionally, it remains to be seen how a heart with more fibrosed myocardium will react to epicardial compression; it is possible that a stiffer myocardium will respond differently to compressive assist; however, it is still anticipated that the heart will perform less work and overall hemodynamics will improve. Finally, the esmolol model lacks arrhythmias that may be present in certain HF etiologies; the CorInnova device triggers with the native ECG, similar to IABP; however, triggering is gated on the R-wave alone with no consideration of P wave. Thus, it is expected that triggering in patients with, for example, atrial fibrillation can be potentially manageable. Specifically, the RR interval is irregular with atrial fibrillation such that RR can be so short that systole is too brief for device activation. Given that the device is non-obligatory in nature, it is expected that a pause in active assist during these periods of rhythm disturbance conditions will permit device use in most patients. Nevertheless, it could be a limitation and will need to be studied more in the future. While a sample size with four animals showed statistically significant changes in CO, LVSV, SBP, MAP, LVP, and LVSW between device standby and assist, more samples from future studies may show statistically significant changes in HR, DBP, CVP, and LVEDP with device activation.

A device-related limitation of significance was the restricted size range of prototypes, and thus, it was possible that the available device(s) for any one study may not have been the ideal size for that particular heart. Preliminary imaging was not completed prior to each study for device sizing; rather, a device was chosen from a set of up to three sizes, based on intraoperative fluoroscopic imaging and by recommendation of the lead surgeon. While the passive component of the device has significant volume and can be adjusted for variations in circumferential dimension, substantially larger hearts were more difficult to accommodate. This was apparent in the animals excluded from data analysis, where the heart was unexpectedly large; these animals experienced acute depression of cardiac function upon deployment. However, additional sizes of the device prototype were available during successive animal studies. Future studies will incorporate a wider range of available device sizes.

## Conclusion

Results of the current study show the potential of the CorInnova device to assist the failing heart acutely. Minimally invasive surgical implantation was a repeated success, combined with significant recovery of cardiac hemodynamics with device assist during a depressed state. Furthermore, the CorInnova device unloads the heart during assist by reducing the amount of ventricular work. The next phase in developing this technology will include longer-term implantation, an investigation of diastolic effects of assist, and a pilot investigation of the CorInnova device in a chronic heart failure model with dilated growth and remodeling.

## Electronic supplementary material

ESM 1 (MP4 34,574 kb)

## Abbreviations

AoP: Aortic pressure
AUP: Animal use protocol
BiV: Biventricular
BTD: Bridge-to-decision
CO: Cardiac output
CPI: Cardiac Power Index
CVP: Central venous pressure
DCC: Direct cardiac compression
ECG: Electrocardiogram
ECMO: Extracorporeal membrane oxygenation
EDP: End diastolic pressure
HF: Heart failure
IABP: Intra-aortic balloon pump
IACUC: Institutional Animal Care and Use Committee
IVC: Inferior vena cava
LA: Left atrium
LV: Left ventricle
LVAD: Left ventricular assist device
LVP: Left ventricular pressure
MCS: Mechanical circulatory support
PA: Pulmonary artery
PTFE: Polytetrafluoroethylene
RA: Right atrium
RV: Right ventricle
SBP: Systolic blood pressure
SV: Stroke volume
SW: Stroke work
VA: Veno-arterial
VAD: Ventricular assist device
W_D_: Device work
W_LV_: Left ventricular work
